# Knockdown of trem2 promotes proinflammatory microglia and inhibits glioma progression via the JAK2/STAT3 and NF-κB pathways

**DOI:** 10.1186/s12964-024-01642-6

**Published:** 2024-05-15

**Authors:** Yunji Yan, Shengwei Bai, Hongxi Han, Junqiang Dai, Liang Niu, Hongyu Wang, Qiang Dong, Hang Yin, Guoqiang Yuan, Yawen Pan

**Affiliations:** 1 Department of Neurosurgery, Lanzhou University Second Hospital, No.82, Cuiyingmen, Chengguan District, Lanzhou City, 730030 Gansu Province China; 2 Department of Neurosurgery, First Affiliated Hospital of Kunming Medical University, Kunming, 650032 Yunnan China; 3 Key Laboratory of Neurology of Gansu Province, Lanzhou University Second Hospital, No.82, cuiyingmen, Chengguan District, Lanzhou City, 730030 Gansu Province China

**Keywords:** Glioma, Glioblastoma, Microglia, Trem2 protein, Stat1 protein, Microglial polarization

## Abstract

**Background:**

In the tumor immune microenvironment (TIME), triggering receptor expressed on myeloid cells 2 (trem2) is widely considered to be a crucial molecule on tumor-associated macrophages(TAMs). Multiple studies have shown that trem2 may function as an immune checkpoint in various malignant tumors, mediating tumor immune evasion. However, its specific molecular mechanisms, especially in glioma, remain elusive.

**Methods:**

Lentivirus was transfected to establish cells with stable knockdown of trem2. A Transwell system was used for segregated coculture of glioma cells and microglia. Western blotting, quantitative real-time polymerase chain reaction (qRT‒PCR), and immunofluorescence (IF) were used to measure the expression levels of target proteins. The proliferation, invasion, and migration of cells were detected by colony formation, cell counting kit-8 (CCK8), 5-ethynyl-2’-deoxyuridine (EdU) and transwell assays. The cell cycle, apoptosis rate and reactive oxygen species (ROS) level of cells were assessed using flow cytometry assays. The comet assay and tube formation assay were used to detect DNA damage in glioma cells and angiogenesis activity, respectively. Gl261 cell lines and C57BL/6 mice were used to construct the glioma orthotopic transplantation tumor model.

**Results:**

Trem2 was highly overexpressed in glioma TAMs. Knocking down trem2 in microglia suppressed the growth and angiogenesis activity of glioma cells in vivo and in vitro. Mechanistically, knockdown of trem2 in microglia promoted proinflammatory microglia and inhibited anti-inflammatory microglia by activating jak2/stat1 and inhibiting the NF-κB p50 signaling pathway. The proinflammatory microglia produced high concentrations of nitric oxide (NO) and high levels of the proinflammatory cytokines TNF-α, IL-6, and IL-1β, and caused further DNA damage and promoted the apoptosis rate of tumor cells.

**Conclusions:**

Our findings revealed that trem2 in microglia plays a significant role in the TIME of gliomas. Knockdown of trem2 in microglia might help to improve the efficiency of inhibiting glioma growth and delaying tumor progression and provide new ideas for further treatment of glioma.

**Supplementary Information:**

The online version contains supplementary material available at 10.1186/s12964-024-01642-6.

## Background

Gliomas are the most common primary malignancy in the brain, and most patients have a poor prognosis and a short survival period, especially those with glioblastoma (GBM). Despite various individualized treatment options, GBM patients have a median survival of only 14–17 months [1]. Evidence that the TIME has a key role in the progression of gliomas, especially TAMs, which present a tumor-promoting phenotype, is accumulating [2]. Therefore, trem2 is a very important factor for understanding the immunosuppressive mechanism of TAMs in glioma for further treatment.

In the central nervous system, trem2 is closely associated with microglial phagocytosis, migration and activation [3]. Overexpression of trem2 in microglia promotes phagocytosis and inhibits the production of inflammatory cytokines, while knockdown of trem2 has the opposite effect [4]. Trem2 is highly expressed mainly in TAMs and has been widely studied in various malignant tumors [5], including breast cancer [6], ovarian cancer [7], hepatocellular carcinoma [8], colorectal cancer [9], clear cell renal carcinoma [10] and lung adenocarcinoma [11]. Many studies have shown that targeting trem2 on TAMs enhances immunotherapy [7] and that trem2 deficiency and trem2 blockade induce a stronger antitumor response in mice [9].

In glioma, upregulated expression of trem2 in microglia creates an immunosuppressive environment that promotes tumor progression and implies a poor prognosis [12]. In addition, trem2 is also related to microglia-induced angiogenesis [13] and the recruitment and response of T cells [12, 14, 15]. Furthermore, downregulation of trem2 in glioma cells resulted in a significant growth-inhibitory effect [16]. Overall, trem2 acts as an important immunomodulatory factor and has great therapeutic potential in glioma, but the detailed molecular mechanisms remain elusive.

NO is an important immunoregulatory signaling molecule and is critical in mediating the signaling communication between microglia and glioma cells [17]. A sufficient concentration of NO can directly induce apoptosis of tumor cells in vivo and in vitro [18, 19]. Previous studies have suggested that knockdown of trem2 increases the levels of lipopolysaccharide (LPS)-stimulated NO [20]. Trem2-knockdown THP-1 cells reduced the invasion ability of glioma cells when cocultured [12]. These studies indicated that knockdown of trem2 in microglia may promote the expression of nitric oxide synthase-2 (Nos2) and reinforce its proinflammatory phenotype.

In this study, we investigated the role of trem2-driven polarization of TAMs in glioblastoma progression. Knockdown of trem2 in microglia promoted an antitumor phenotype through activation of the jak2/stat1 and NF-κB p50 signaling pathways, releasing high levels of proinflammatory cytokines and high concentrations of NO. Cytotoxic NO resulted in elevated levels of ROS, which in turn induced DNA damage, ultimately promoting apoptosis of tumor cells.

## Methods and materials

### Glioma samples and ethical approval

For the Western blotting assay, 24 human glioma samples and 8 brain tissues from patients who suffered from brain trauma were collected from the Second Affiliated Hospital of Lanzhou University. Twelve GBM tissues, 6 traumatic brain tissues and 6 normal brain tissues were used for the IF experiment. We obtained normal brain tissues from patients who were diagnosed with deep brain GBM and had to undergo fistula surgery. This study was approved by the Clinical Ethics Committee of the Second Hospital of Lanzhou University.

### RNA extraction and qRT–PCR

The M5 Universal RNA Mini Kit (Mei5bio, Beijing, China) was used to extract total RNA from glioma cells. The riboSCRIPT mRNA/lncRNA qRT‒PCR Starter Kit (RiboBio, Guangzhou, China) was used to perform qRT–PCR assays on the CFX96 Touch Real-Time PCR Detection System (Bio-Rad, USA). The primer sequences were as follows: Trem2, F 5’- ACAGAAGCCAGGGACACA TC-3’, R 5’- CCTCCCATCATCTTCCTTCA-3’; Gapdh, F 5’- GCCATCACAGCAACACAGAA-3’, R 5’- GCCATACCAGTAAGCTTGCC-3’.

### Cell line culture

BV2 and HMC3 microglial cell lines and U-87MG and U-251MG glioma cell lines were purchased from the American Type Culture Collection cell bank. The Gl261 and HUVEC lines were a kind gift from Cheng Jiang (Huazhong University of Science and Technology, China). HMC3 microglia were cultured using minimum Eagle’s medium (MEM, Solarbio, Beijing, 41,500), penicillin–streptomycin (Sangon Biotech, Shanghai, China, E607011), and 10% fetal bovine serum (FBS, Sangon Biotech, E510008). BV2, U-87MG, U-251MG and Gl261 cells were cultured using Dulbecco’s modified Eagle’s medium (DMEM, Solarbio, 11,995). For the glutamine deprivation assay, cells were cultured in glutamine-free culture medium. HUVECs were cultured using endothelial cell medium (ECM, ScienCell, USA, 1001).

### Transfection

Four shRNA sequences were purchased from HanBio (www.hanbio.net). The sequences were as follows: shRNA-NC: 5′-TTCTCCGAACGTGTCACGTAA‐3′, shRNA‐1: 5′‐GAGCCTCTTGGAAGGAGAAAT‐3′, shRNA‐2: 5′‐CACAGCCATCACAGACGATAC‐3′, and shRNA‐3: 5′‐CTCACCATTACGCTGCGGAAT‐3′. Puromycin (2 µg/mL) was used to establish stable cell lines after transfection with lentivirus.

### EdU, CCK-8 and colony formation assays

The EdU Cell Proliferation Kit (Sangon Biotech, E607204) was used for the EdU assay according to the operational instructions. For the CCK-8 assay, 10% CCK-8 reagent was added to the medium of each well of the plates (96-well), and after incubation for 2 h, the absorbance was measured by a microplate reader (Bio-Tek, USA). For the colony formation assay, 1000 cells were added to each well of a plate (6 wells) and cultured for 2 weeks in a cell incubator. After washing with PBS and fixing with 4% paraformaldehyde, the plates were stained with crystal violet.

### Transwell assay

After coculture, transwell assays were performed with Transwell 24-well plates (Corning, USA, 3422). Then, 700 µL of complete medium was added to the wells of a 24-well plate, and 100 µL of serum-free medium with 3.0 × 10^4^ cells was placed into the upper chambers. Matrigel solution (Corning, USA, 356,234) was used for the invasion assay.

### Flow cytometry analysis

An Annexin V-FITC/PI Apoptosis Detection Kit (Yeasen, Shanghai, 40,302) was used to assess the apoptosis ratio of the cells. The cell cycle distribution of cells was detected by using a Cell Cycle Staining Kit (MultiSciences, Hangzhou, China, CCS012). The intracellular ROS level of target cells was measured by a Reactive oxygen species Assay Kit (Jiancheng Bioengineering, Nanjing, China, E004-1-1). All flow cytometry analyses were performed with a CytoFLEX S (Beckman, USA). All fcs data were analyzed with FlowJo V10 or ModFit LT.

### Alkaline comet assay

The first layer of gel was formed by 0.8% normal melting point agarose. After sufficient solidification, the second layer of gel was formed by 25 µl of PBS containing 1000 cells and 75 µl of 0.8% low melting point agarose. Cells were subsequently lysed in 4 °C comet assay lysis buffer for 3 h. Then, the slide was allowed to unwind in alkali buffer for 30 min before electrophoresis. Finally, the cells were neutralized with 0.4 M tris(hydroxymethyl)aminomethane and stained with 4’,6-diamidino-2-phenylindole (DAPI). Data analysis was conducted using casp_1.2.3b1 software.

### Tube formation assay

After coculture with different groups of microglia, glioma cells were further cocultured with HUVECs for 48 h. Subsequently, the HUVECs were resuspended in coculture conditioned medium (CM) for the tube formation assay. Each well of a 96-well plate was precoated with 50 µl of Matrigel solution (Corning, 356,234) and then incubated in a cell incubator for 30 min to allow solidification. HUVECs (2 × 10^4^) were then resuspended in 200 µl of CM and cultured for 6 h before image acquisition.

### Detection and removal of NO

For the individual culture group, 2 × 10^5^ HMC3 or glioma cells in 1 ml DMEM were seeded into each well of 24-well plates. For the cocultured group, 1 × 10^5^ HMC3 and 1 × 10^5^ glioma cells were suspended in 1 ml DMEM and added to 24-well plates. After 48 h, NO production in cells was measured by the Griess method, as indicated in the NO assay kit (Beyotime, Jiangsu, China, S0021S). Carboxy-PTIO (100 µM, Beyotime, S1546) was used to remove NO from the culture medium.

### Western blotting

Radioimmunoprecipitation assay (RIPA, Beyotime, P0013K) buffer containing protease inhibitors and/or phosphatase inhibitors was used to extract proteins from cells and glioma tissues. A membrane and cytosol protein extraction kit (Beyotime, P0033) was used to extract membrane proteins from cultured cells. After sufficient centrifugation and boiling for denaturation, protein samples were isolated and then electrotransferred onto PVDF membranes. After blocking with 5% bovine serum albumin (BSA, Beyotime, ST023-50 g), membranes were successively soaked in diluted primary and secondary antibody solutions. Finally, membranes were visualized with ImageQuant LAS 500 (GE, USA). Primary antibodies included rabbit anti-trem2 (1:500, Abcam, ab209814), rabbit anti-bax (1:1000, Proteintech, Wuhan, China, 50599-2-Ig), rabbit anti-bcl-2 (1:1000, Proteintech, 12789-1-AP), rabbit anti-caspase-3 (1:1000, Abcam, ab184787), rabbit anti-cd11b (1:1000, Abcam, ab133357), mouse anti-gapdh (1:40000, Proteintech, 60004-1-Ig), rabbit anti-γH_2_ax (1:5000, Abcam, ab81299), rabbit anti-il-1β (1:500, Bioss, Beijing, bs-0812R), rabbit anti-il-6 (1:500, Bioss, bs-6309R), rabbit anti-il-10 (1:1000, Proteintech, 20850-1-AP), rabbit anti-iNOS (1:500, Affinity, Changzhou, China, AF0199), rabbit anti-jak2 (1:500, Proteintech, 17670-1-AP), rabbit anti-NF-κB p105/p50 (1:2000, Abmart, Shanghai, M005873), rabbit anti-Phospho-jak2 (1:500, Abmart, T56570), rabbit anti-cleaved-caspase-3 (1:500, Abmart, TA7022), rabbit anti-Phospho-stat1 (1:500, Abmart, TP56498), rabbit anti-Phospho-stat3 (1:500, Affinity, AF3293), rabbit anti-stat1 (1:500, Abmart, T55227), mouse anti-stat3 (1:1000, Proteintech, 60199-1-Ig), rabbit anti-TNF Alpha (1:500, Proteintech, 17590-1-AP), mouse anti-vegfa (1:1000,Preteintech, 66828-1-Ig), rabbit anti-ATP1A1 (1:5000, Proteintech, 14418-1-AP). HRP Goat Anti-Rabbit IgG (H + L) (1:4000, Abclonal, Wuhan, AS014), HRP Goat Anti-Mouse IgG (H + L) (1:4000, Abclonal, AS003) were used as secondary antibodies.

### Mice and tumor model

8-week-old male C57BL/6 mice were provided by the Animal Center of Lanzhou University. TREM2 homozygous knockout (Trem2^−/−^) mice was purchased from Southern Medical University of china and 8 8-week-old male trem2^−/−^ mice were used for tumor model. For the intracranial tumor model, mice were anesthetized with isoflurane (RWD, Shenzhen, China). Once surgical anesthesia was finished, mice were placed into a digital stereotaxic instrument (RWD). After fully exposing the skull at the injection site coordinates (+ 0.7 mm anterior, -2.0 mm lateral from bregma), gl261 cells (2 × 10^5^) were slowly injected in a volume of 5 µl at a depth of 3 mm and left in place for 10 min. The mice were euthanized on the 12th day after injection, and brain tissues were extracted.

### IF and IHC

Tumor samples were fixed with paraformaldehyde for at least 1 week and then dehydrated by a Fully Enclosed Tissue Processor (Leica, ASP300) before embedding in paraffin. Paraffin Sect. (4 μm) were dewaxed with different concentrations of xylene and ethanol. After antigen repair and blocking with 5% normal goat serum in PBS for 1 h at room temperature, the tissues were treated with an antibody. After incubation with specific IF labeling reagents, the slides were observed promptly. The cell slides were additionally permeabilized with 0.1% Triton X-100 (Sigma) in PBS for 10 min before blocking. Fluorescence images were captured by a Leica DM4B microscope, Zeiss LSM880 confocal laser scanning microscope, or TissueFAXS Plus quantitative imaging system. For hematoxylin and eosin staining, slides were first placed in Harris’s hematoxylin solution (Biosharp, BL700A), washed with PBS for seconds and finally stained with 1% alcoholic eosin (Biosharp, BL700A).

Primary antibodies included mouse anti-IBA1(1:400, Servicebio, Wuhan, GB12105), rabbit anti-trem2 (1:50, Thermofisher, USA, PA5-87933), rabbit anti-γH_2_ax (1:200, Abcam, ab81299), rabbit anti-CD105 (1:200, Bioss, bs-0597R), rabbit anti-Ki-67 (1:300, Affinity, AF0198). Goat Anti-Rabbit IgG H&L (Alexa Fluor® 488) (1:200, Abcam, ab150077), Goat Anti-Mouse IgG H&L (Alexa Fluor® 488) (1:200, Abcam, ab150113), Goat Anti-Rabbit IgG H&L (Alexa Fluor® 594) (1:200, Abcam, ab150080), Goat Anti-Mouse IgG H&L (Alexa Fluor® 594) (1:200, Abcam, ab150116) were used as secondary antibodies.

### Statistical analysis

All data were handled by GraphPad Prism 8.0.1. All results of at least three independent trials are reported as the means ± SD. Differences between groups were evaluated using Student’s t test. Differences with *P* values < 0.05 were set as statistically significant.

## Results

### Trem2 was upregulated in human glioma and mouse glioma tissues

**Fig. 1:**
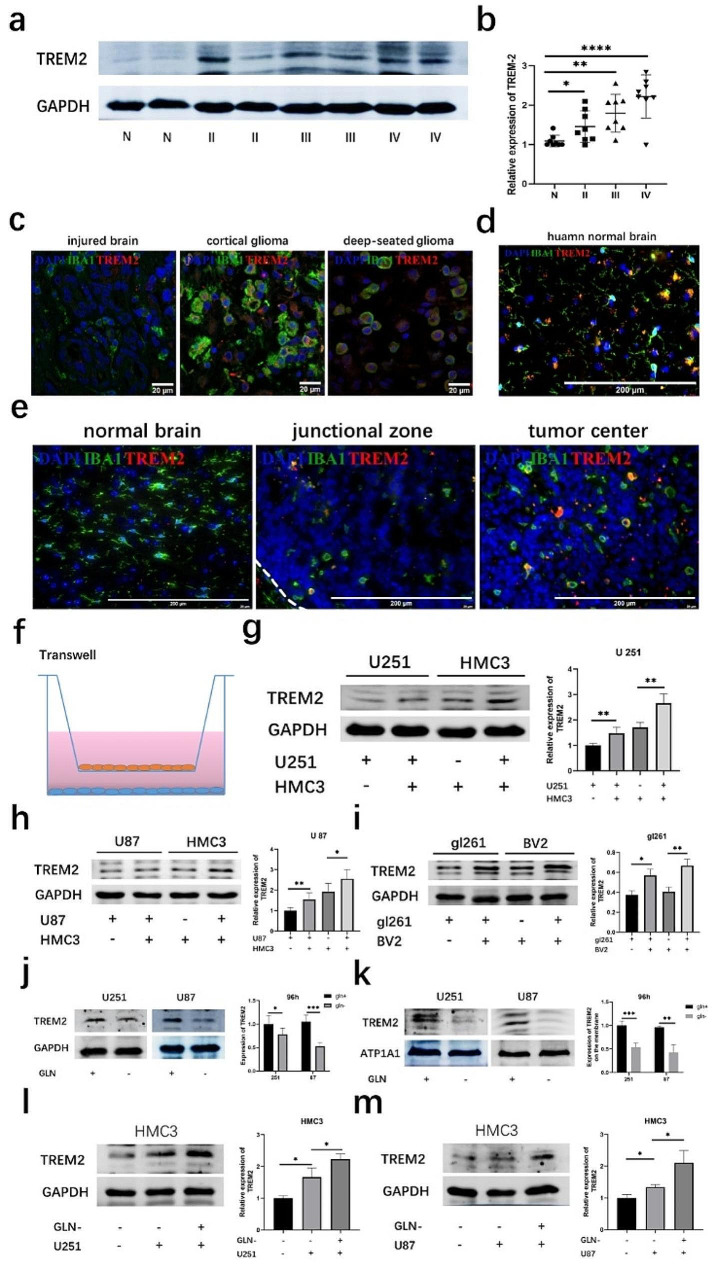
Trem2 was upregulated in human glioma and mouse glioma tissues. **(a-b)** Western blotting assay of trem2 expression levels in injured human brain tissues and glioma tissues of different WHO tumor grades (*n* = 8 in each group). **(c)** Trem2 was highly overexpressed in tumor cells and microglia in both cortical and deep-seated GBM tissues, while it was barely expressed in injured brain tissue (*n* = 6 in each group). **(d)** In normal human brain tissues, microglia appear in a resting state, with many branches, and do not express trem2 (*n* = 6). **(e)** Trem2 was not expressed in the normal mouse brain and was highly expressed in glioma. Compared to that in the junctional zone of mouse glioma, trem2 was more highly expressed in the core zone of the tumor (*n* = 6 in each group). **(f)** Diagram showing a Transwell segregated coculture setup. **(g)** Western blot assays were performed to detect the expression of trem2 in U251 cells and HMC3 cells. **(h)** Expression level of trem2 in U87 cells and HMC3 cells. **(i)** Expression level of trem2 in gl261 cells and BV2 cells. **(j)** Trem2 in glioma was downregulated after glutamine deprivation for 96 h. **(k)** Trem2 of membrane proteins in glioma cells was downregulated after glutamine deprivation for 96 h. **(l-m)** Glutamine-deprived glioma cells were more capable of upregulating trem2 expression in microglia when cocultured

To clarify the role of trem2 in glioma, the expression level of trem2 was examined in different grades of glioma tissues, with the injured brain as its control. The results showed that trem2 was highly expressed in glioma tissues and gradually increased with increasing tumor grade, which was followed the World Health Organization (WHO) tumor classification system (Fig. 1a-b). We further detected the expression of trem2 in different cell types by IF experiments. We found that trem2 was highly overexpressed in glioma cells and microglia in both cortical and deep-seated GBM tissues, while it was barely expressed in injured brain tissue (Fig. 1c). Microglia are clearly activated in the injured brain tissues. To exclude the impact of traumatic stimulation on trem2 expression in microglia, we further examined the expression level of trem2 in normal brain tissue from patients with deep brain gliomas who had to undergo fistula surgery. The results suggested that in completely normal human brain tissues, microglia appear in a resting state, with many branches, and do not express trem2 (Fig. 1d). Then, we further explored the expression level of trem2 in mouse glioma tissues. As in human brain tissue, trem2 was not expressed in normal mouse brain tissues but was highly expressed in mouse glioma tissue, mainly on microglia (Fig. 1e). Furthermore, compared to that in the junctional zone of mouse glioma, trem2 was highly overexpressed in the core zone of the tumor (Fig. 1e). The above experimental results indicated that the expression of trem2 in glioma tissues may play an important role in glioma progression.

### In the coculture model, trem2 was upregulated in both microglia and glioma cells

To formally investigate the role of trem2 in the TIME, a segregated coculture Transwell setup (Fig. 1f) was utilized to assess the expression of trem2 [17]. Trem2 in human glioma cells and human microglia was upregulated following segregated coculture with each other compared to that following coculture with themselves (Fig. 1g-h). We repeated the above coculture experiments with BV2 murine-derived microglia and gl261 murine-derived tumor cells and came to the same conclusion (Fig. 1i). Collectively, these findings indicated that microglia highly expressed trem2 in the TIME and promoted the malignant progression of glioma.

### Glutamine deprivation downregulates the expression of trem2 in tumor cells

As previous results (Fig. 1e) showed, the expression of trem2 varied in the center and junction areas of the glioma. This difference reminds us of the distributional difference of glutamine in glioma tissues. Studies have demonstrated that there is a significant downregulation of glutamine concentrations in the core regions compared to that of those in the tumor periphery [21]. Furthermore, decreased levels of glutamine in the core region have been found to enhance the tumor’s ability to evade the immune system [21]. Therefore, we intend to further explore the correlation of glutamine deficiency with trem2. In glioma cells, trem2 was downregulated after treatment with glutamine deprivation (Fig. 1j). To further demonstrate this correlation, we extracted membrane proteins from glioma cells and showed that glutamine deprivation caused a reduction in trem2 in glioma cells (Fig. 1k). Furthermore, when we cocultured glioma cells after glutamine deprivation with microglia, we found that glutamine-deprived glioma cells were more capable of upregulating trem2 expression in microglia (Fig. 1l-m). These results suggested that the immune escape of glioma cells caused by glutamine deprivation was possibly related to the altered expression levels of trem2.

### Knockdown of trem2 enhanced the microglial antitumoral phenotype in vitro

**Fig. 2:**
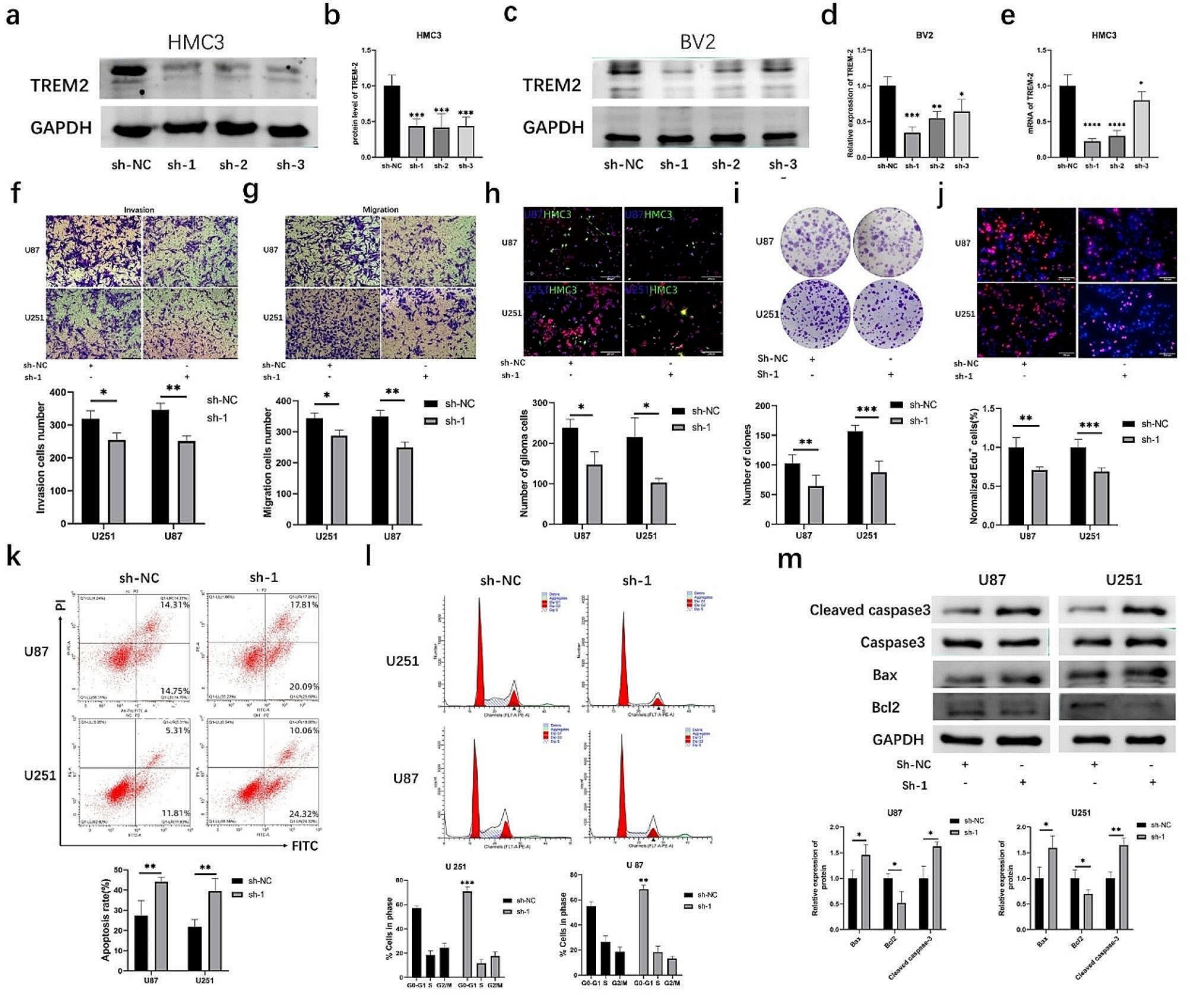
Knockdown of trem2 enhanced the microglial antitumoral phenotype in vitro. **(a-d)** The protein expression of trem2 in HMC3 and BV2 cells. **(e)** qRT‒PCR assay was performed to detect the expression of trem2 in HMC3 cells. **(f-g)** Transwell assays showed that trem2-knockdown microglia suppressed the cell migration and invasion ability of glioma. **(h)** Trem2-knockdown microglia inhibited tumor growth of human glioma cells were directly cocultured with HMC3 cells. **(i)** A colony formation assay was performed to detect cell proliferation. **(j)** Cell proliferation abilities were assessed by EdU assay. **(k-l)** Flow cytometry analysis showed that trem2-knockdown microglia arrest the glioma cell cycle at the G1 phase and promote the apoptosis rate of glioma cells. **(m)** Western blot analysis showed elevated expression of apoptotic proteins and decreased expression of anti-apoptotic proteins

To delve deeper into the biological impact of trem2 in the TIME, we successfully developed a lentiviral-based stable interference system for trem2 (sh-trem2). Western blot assays demonstrated a significant reduction in trem2 expression in both HMC3 and BV2 cells (Fig. 2a-d). Additionally, qRT‒PCR assays confirmed the effect of knockdown in HMC3 cells (Fig. 2e). After knocking down trem2 in microglia, we found that glioma cells cocultured with trem2-knockdown microglia cells exhibited a notably reduced migration and invasion ability (Fig. 2f-g). To observe the interaction of microglia and glioma cells, U87-mCherry or U251-mCherry human glioma cells were directly cocultured with HMC3-GFP cells at a density of 7:1 as reported [22]. The results showed that trem2-knockdown microglia inhibited tumor growth of glioma cells (Fig. 2h), and this inhibition was also observed in separate coculture conditions (Fig. 2i-j). Furthermore, we also performed flow cytometry analysis, which indicated that trem2-knockdown microglia arrested the glioma cell cycle at the G1 phase and promoted the apoptosis rate of glioma cells (Fig. 2k-l). Western blot analysis also showed that trem2-knockdown microglia increased cleaved caspase-3 and bax protein levels but decreased bcl-2 protein levels in glioma cells (Fig. 2m). These results indicated that knockdown of trem2 remodeled the phenotype of microglia under coculture conditions and exerted a tumor-suppressor effect.

### Trem2-knockdown microglia produce high concentrations of cytotoxic nitric oxide and DNA damage

**Fig. 3:**
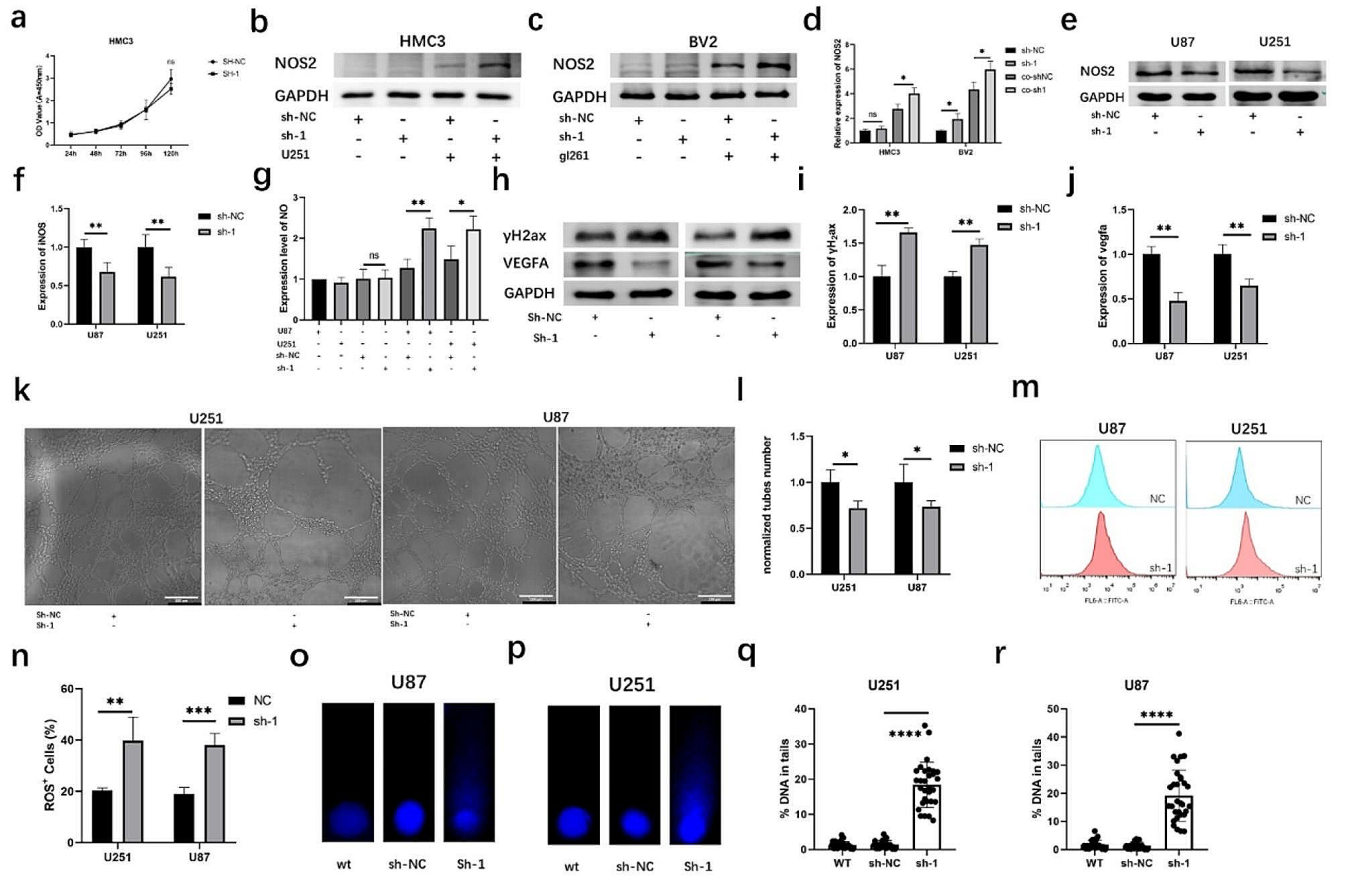
Trem2-knockdown microglia produce high concentrations of cytotoxic nitric oxide and DNA damage. **(a)** CCK-8 assay showed that knockdown of trem2 in HMC3 did not affect the cell growth of microglia. **(b-d)** Western blot analysis of Nos2 in HMC3 and BV2 cells. **(e-f)** Western blot analysis of Nos2 in U87 and U251 cells. **(g)** The concentration of NO in the culture medium. **(h-j)** Western blot analysis of VEGFA and γH_2_ax in U87 and U251 cells. **(k-l)** Tubule formation assay showed the angiogenesis ability of glioma cells. **(m-n)** Flow cytometry analysis showed the intracellular ROS levels in U87 and U251 cells. **(o-r)** The alkaline comet assay showed the level of DNA damage in glioma cells

In the TIME, microglia can acquire an anti-inflammatory phenotype, which is associated with a tumor-promoting effect, and secrete anti-inflammatory cytokines, such as IL-10. Alternatively, microglia can acquire an proinflammatory phenotype, characterized by an antitumor role in the TIME and release proinflammatory cytokines such as IL-1β, IL-6, TNF-α, ROS, and NO [23]. When microglial trem2 was knocked down, microglial growth was not affected (Fig. 3a). To further explore the mechanism underlying the tumor-suppressive effect of trem2-knockdown microglia, we measured the expression of Nos2 and found that Nos2 was upregulated in trem2-knockdown microglia (Fig. 3b-d). Published studies have provided evidence that glioma cells can produce low concentrations of NO and drive microglia to express an anti-inflammatory phenotype to promote tumor growth [17]. Therefore, we further detected Nos2 in glioma cells and found that Nos2 was downregulated after coculture with trem2-knockdown microglia (Fig. 3e-f). Then, we detected the production of NO in the culture medium and found that the medium in which trem2-knockdown microglia were directly cocultured with glioma cells had higher concentrations of NO (Fig. 3g). High concentrations of nitric oxide could produce large amounts of highly cytotoxic ROS and hinder DNA repair through multiple mechanisms [24]. Therefore, we further detected the role of trem2-knockdown microglia in vascularization and DNA damage in glioma. As shown in the figure, the expression level of γH_2_ax was upregulated and that of VEGFA was downregulated in glioma (Fig. 3h-j). This tumor angiogenesis inhibition was also confirmed by the tubule formation assay (Fig. 3k-l). The intracellular ROS level was also upregulated in glioma cells (Fig. 3m-n). The alkaline comet assay proved that the level of DNA damage in glioma cells cocultured with trem2-knockdown microglia was higher (Fig. 3o-r). These experimental results demonstrated that trem2-knockdown microglia inhibit glioma growth by producing high concentrations of cytotoxic nitric oxide, which causes higher ROS levels and DNA damage while inhibiting the vascularization of glioma.

### Effect of trem2 on STAT1 phosphorylation and the NF-κB p50 signaling pathway

**Fig. 4:**
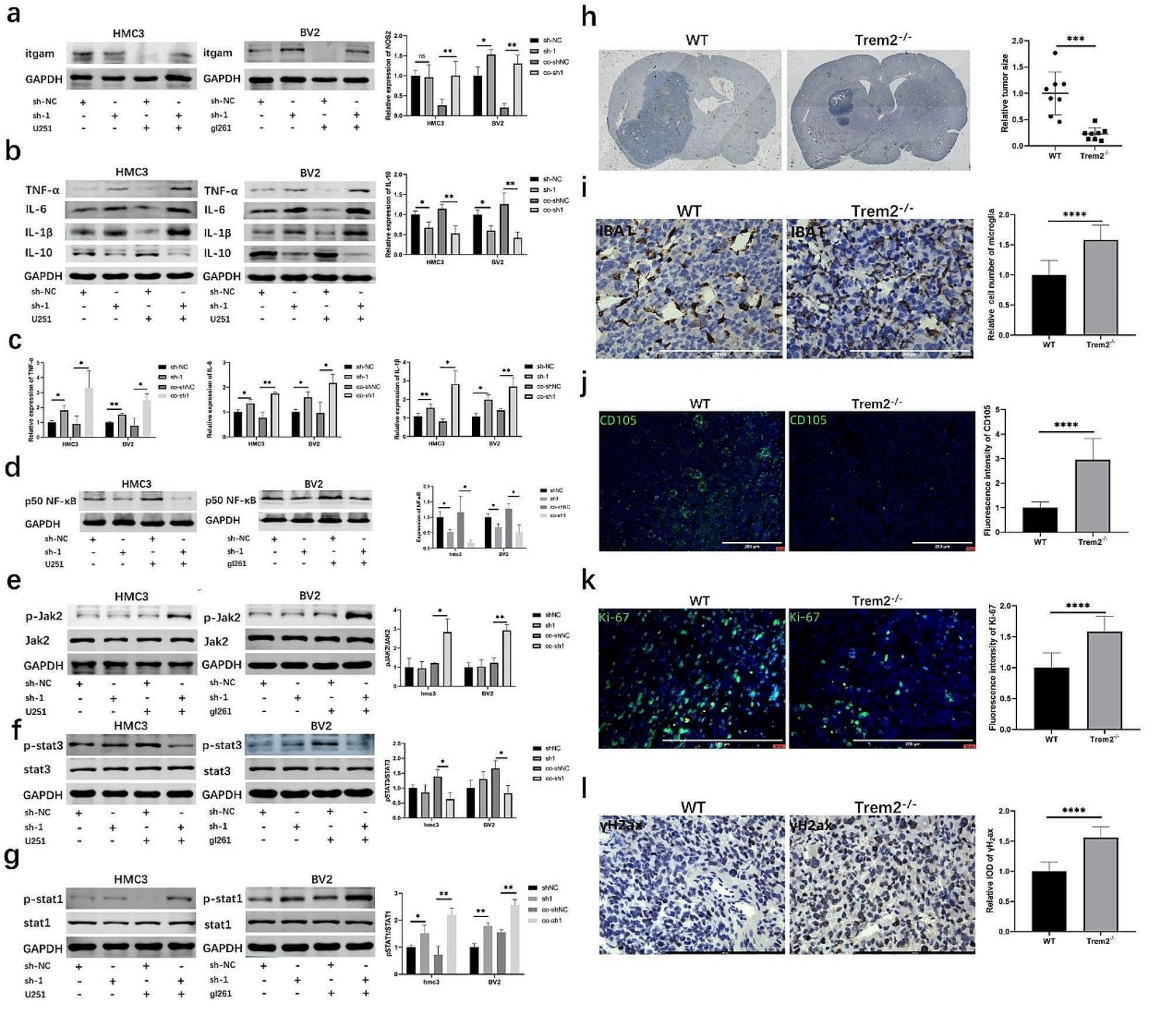
Effect of trem2 on STAT1 phosphorylation and the NF-κB p50 signaling pathway, as well as animal studies. **(a)** Western blot analysis of itgam in HMC3 and BV2 cells. **(b-c)** Western blot analysis of anti-inflammatory cytokines (IL-10) and pro-inflammatory cytokines (IL-1β, IL-6, TNF-α). **(d)** Western blot analysis of NF-κB p50 in microglia. **(e)** Western blot analysis of jak2 and p-jak2 in microglia. **(f)** Western blot analysis of stat3 and p-stat3 in microglia. **(g)** Western blot analysis of stat1 and p-stat1 in microglia. **(h)** Representative image of intracranial tumor of mice (*n* = 8 of each group). **(i)** The representative IHC images of IBA1. **(j)** The representative IF images of CD105. **(k)** The representative IF images of Ki-67. **(l)** The representative IHC images of γH_2_ax

Itgam is considered an activation marker of microglia [25, 26] and the microglial proinflammatory phenotype is mainly characterized by overexpression of itgam [27]. In this study, we found that the expression of itgam in trem2-knockdown microglia was upregulated when cocultured with glioma cells compared to that in the control (Fig. 4a). Researches proved that proinflammatory macrophages can promote inflammatory responses by activating the Jak2/Stat1 pathway, secreting cytokines TNF-α, IL-1α, IL-1β, IL-6, IL-12, and high levels of NO. Additionally, they can also promote the anti-inflammatory phenotype through the IL-10-mediated Jak2/Stat3 pathway [28]. Then, we detected anti-inflammatory cytokines and proinflammatory cytokines in trem2-knockdown microglia and found that IL-1β, IL-6, and TNF-α were upregulated, while IL-10 was downregulated (Fig. 4b-c). Studies have suggested that the p50 subunit of NF-κB activates the secretion of inflammatory genes and may shift cell polarization from proinflammatory to anti-inflammatory [29]. Therefore, we further measured the expression levels of Jak2/p-Jak2, stat3/p-stat3, stat1/p-stat1, and NF-κB p50 in trem2-knockdown microglia, and the results showed that the levels of p-Jak2 and p-stat1 were significantly increased, while the levels of p-STAT3 and NF-κB p50 were reduced (Fig. 4d-g). Taken together, these results demonstrate that trem2-knockdown microglia play a proinflammatory role when cocultured with glioma cells. Trem2-knockdown microglia produced high concentrations of NO and released proinflammatory cytokines through the jak2/stat1 and NF-κB p50 signaling pathways when cocultured with glioma cells.

### Trem2 knockout can inhibits growth of glioma cells in vivo

To further elucidate the tumor growth inhibitory effect of trem2 knockout microglia, we employed an in vivo experimental glioma model with WT or trem2^−/−^ mice. By establishing an in situ glioma model with gl261 cells, we observed a significant inhibition of tumor growth in trem2^−/−^ mice compared to WT mice (Fig. 4h). Compared to wild-type mice, the number of TAMs in trem2^−/−^ mice significantly increased, accompanied by noticeable changes in cell morphology (Fig. 4i). This clearly demonstrated the importance of trem2-knockout microglia in tumor suppression. CD105 was considered as a specific marker of newly formed and activated small blood vessels [30]. Studies suggested that CD105 may be a more suitable marker for measuring microvessel density than CD31 or CD34 [31]. In our study, trem2^−/−^ mice significantly inhibited the expression of Ki-67 compared to WT mice, with a pronounced inhibitory effect on tumor angiogenesis (Fig. 4j-k). Additionally, consistent with previous experimental results, trem2^−/−^ mice showed a more significant DNA damage level in glioma cells (Fig. 4l).

### Nitric oxide scavengers can partially rescue the tumor growth inhibition of Trem2 knockdown microglia

**Fig. 5:**
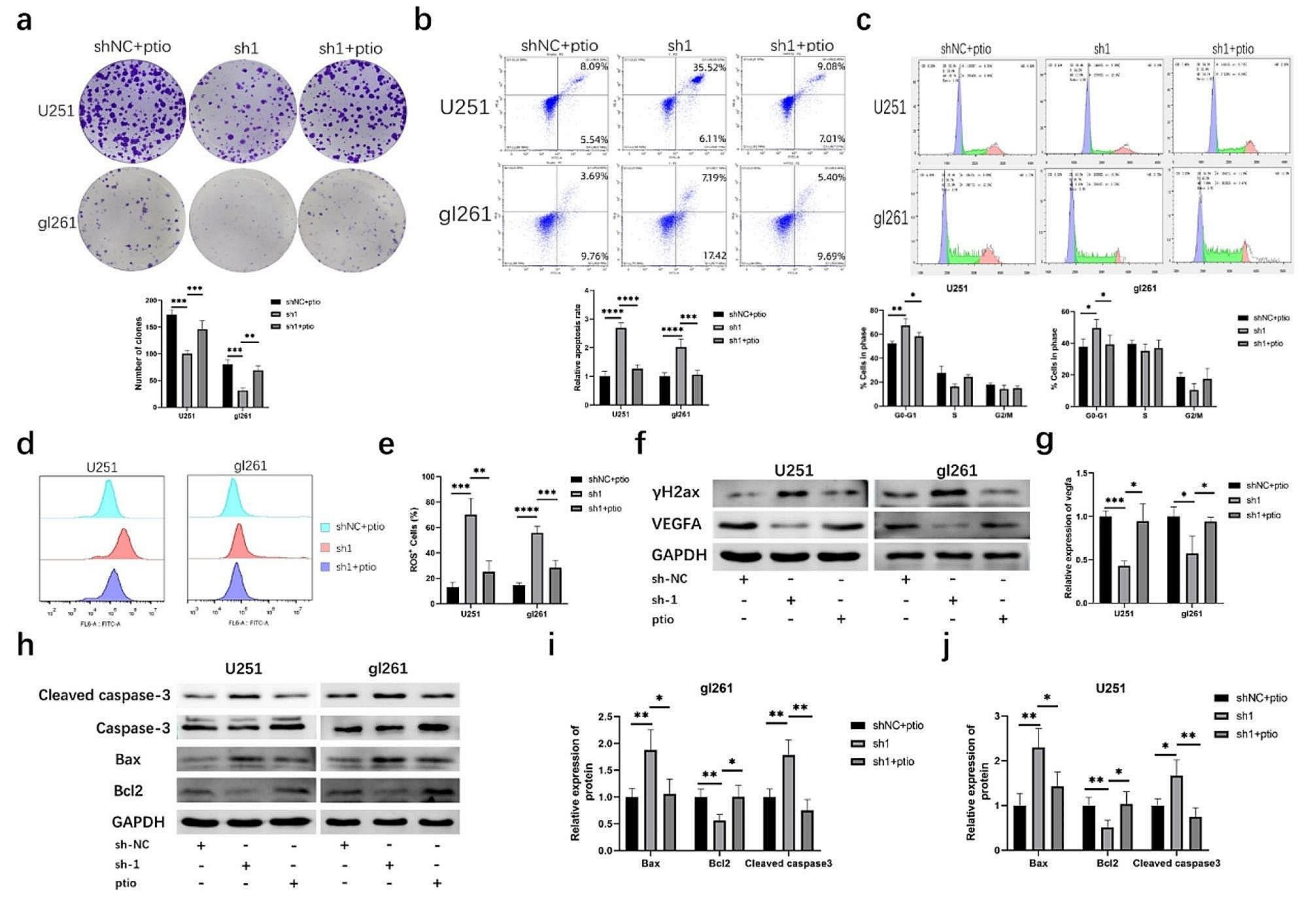
Nitric oxide scavengers can partially rescue the tumor growth inhibition of Trem2 knockdown microglia. **(a)** Colony formation assay showed that the nitric oxide scavenger carboxy-PTIO can partially rescued the tumor growth inhibition of Trem2 knockdown microglia. **(b)** Flow cytometry analysis showed that carboxy-PTIO can partially rescued the promotion of apoptosis rate caused by Trem2 knockdown microglia. **(c)** Flow cytometry analysis was used to detect cell cycle. **(d-e)** Flow cytometry analysis was used to detect the ROS levels in glioma cells. **(f-g)** Western blot analysis of cleaved caspase-3, bax and bcl-2 in glioma cells. **(l-p)** Western blot analysis of VEGFA and γH_2_ax in glioma cells

To further clarify the role of nitric oxide in the coculture system, we used carboxy-PTIO to remove nitric oxide from the culture medium. The colony formation assay showed that carboxy-PTIO rescued the inhibition of tumor growth caused by trem2-knockdown microglia (Fig. 5a). Flow cytometry assays showed that carboxy-PTIO rescued cell cycle arrest and promoted the apoptosis rate caused by trem2-knockdown microglia (Fig. 5b-c). The upregulated ROS level could also be rescued by carboxy-PTIO (Fig. 5d-e). Then, a Western blot analysis was performed to detect the expression of VEGFA and γH_2_ax, and the results also showed that carboxy-PTIO rescued the DNA damage level and inhibitory effect of tumor vascularization (Fig. 5f-g). Furthermore, we detected the expression of apoptotic proteins and anti-apoptotic proteins and found that carboxy-PTIO can clear the increasion in apoptotic proteins and reduction in anti-apoptotic proteins (Fig. 5h-j). These results reveal that NO produced by microglia plays an important tumor-suppressive role in the TIME; when NO was removed, this tumor inhibitory effect of proinflammatory microglia was partially weakened.

## Discussion

TAMs play an important role in the TIME and usually exhibit an anti-inflammatory phenotype that promotes tumor progression [32]. Glioma cells can secrete multiple factors to attract TAMs to the tumor tissue, and then microglia transform into the anti-inflammatory phenotype and release multiple other factors to promote the proliferation and/or migration of glioma [33–35]. In the immune microenvironment of gliomas, myeloid lineage cells are the predominant immune population and can constitute up to 30–50% of the total cellular composition [2]. Further study showed that resident microglia, rather than peripheral macrophages, are the main cell type of TAMs in glioma [36]. Therefore, we mainly focused on the effects of microglia rather than monocyte-derived macrophages from blood on glioma.

NO is a very important immunomodulatory molecule in the TIME and is one of the key molecules mediating the cytotoxicity of glioma cells activated by conditioned medium from microglia [37]. Crosslinking of trem2a on the surface of macrophages leads to the release of NO [38]. Studies have suggested that trem2-negative microglia are more activated than trem2-expressing microglia; in vitro, exposure to LPS/IFNγ dramatically reduced microglial expression of trem2 [39]. Knockdown of trem2 in BV2 cells increased LPS-stimulated NO production [20, 40]. These results enhanced the relationship between trem2 and glioma cytotoxic NO. In this study, we found that after microglial trem2 was knocked down, the release of NO was upregulated, which caused higher ROS levels and DNA damage while inhibiting the vascularization of glioma. Furthermore, we used carboxy-PTIO to remove nitric oxide from the culture medium and proved that NO is an important toxic molecule in glioma cells produced by trem2-knockdown microglia.

Glutamine metabolism plays a crucial role in regulating macrophage activation and the synthesis and secretion of pro-inflammatory cytokines. Previous studies proved that α-ketoglutarate (α-KG) derived from glutamine metabolism induces macrophage reprogramming toward an anti-inflammatory polarized state [41]. Meanwhile, NO is a key determinant for the disruption of the tricarboxylic acid cycle between citric acid and α-KG [42]. These results demonstrate the relationship between macrophage polarization and glutamine metabolism. In this study, a glutamine deprivation assay indicated that the immune escape of glioma cells, caused by glutamine deprivation, was possibly related to the expression of trem2.

Activation of itgam molecules promotes proinflammatory macrophage polarization. Itgam was proven to be closely associated with blood vessel stability in tumors. In animal experiments, itgam^−/−^ tumors showed fewer and longer blood vessels than control tumors [43]. In our study, itgam was upregulated when trem2-knockdown microglia were cocultured with glioma cells. In addition, Nos2 has long been considered a hallmark of proinflammatory polarization in microglia and was also significantly upregulated. These results indicate that trem2-knockdown microglia shift cells from anti-inflammatory to proinflammatory when cocultured with glioma cells and release various proinflammatory cytokines to inhibit the progression of glioma.

Stat1 is considered a major pathway involved in proinflammatory macrophage polarization and results in tumoricidal functions, meanwhile stat3 is considered a key inducer of anti-inflammatory gene transcription [44–48]. Numerous studies have extensively demonstrated that by activating the Jak2/Stat1 pathway in TAMs or inhibiting the Jak2/Stat3 pathway, could shift TAMs from anti-inflammatory state towards proinflammatory activation [49–51]. This strengthens anti-tumor immunity and consequently inhibits tumor progression. NF-κB is a critical modulator of inflammation and remission, and studies have shown that the p50 subunit of NF-κB is a key regulatory factor for anti-inflammatory-driven inflammatory responses both in vivo and in vitro [29, 52]. To further explore the potential mechanism, we detected the changes in the expression of the abovementioned proteins. The results indicated that trem2-knockdown microglia may remodel the cellular phenotype of microglia through the jak2/stat1 and NF-κB pathways and subsequently inhibit tumor growth.

## Conclusion

Our findings revealed that trem2 could remodel the cellular phenotype of microglia to shift cells from anti-inflammatory to proinflammatory. Trem2-knockdown microglia produce high concentrations of NO and various proinflammatory cytokines to inhibit the progression and angiogenesis of glioma through the jak2/stat1 and NF-κB pathways when cultured with glioma cells.

### Electronic supplementary material

Below is the link to the electronic supplementary material.

Supplementary Material 1

## Data Availability

No datasets were generated or analysed during the current study.

## Abbreviations

α-KG: α-ketoglutarate
BSA: Bovine Serum Albumin
CCK8: Cell Counting Kit-8
DAPI: 4’,6-diamidino-2-phenylindole
DMEM: Dulbecco’s modified Eagle’s medium
ECM: endothelial cell medium
EdU: 5-ethynyl-2’-deoxyuridine
FBS: foetal bovine serum
LPS: Lipopolysaccharide
MEM: minimum Eagle’s medium
NO: nitric oxide
Nos2: nitric oxide synthase-2
qRT-PCR: quantitative real-time polymerase chain reaction
RIPA: radio-immunoprecipitation assay
ROS: reactive oxygen species
TAMs: tumor-associated macrophages
TIME: tumor immune microenvironment
Trem2: triggering receptor expressed on myeloid cells 2
WHO: World Health Organization

## References

[1] Molinaro AM, Taylor JW, Wiencke JK (2019). Genetic and molecular epidemiology of adult diffuse glioma. Nat Rev Neurol.

[2] Wei J, Chen P, Gupta P (2020). Immune biology of glioma-associated macrophages and microglia: functional and therapeutic implications. Neuro Oncol.

[3] Mazaheri F, Snaidero N, Kleinberger G (2017). TREM2 deficiency impairs chemotaxis and microglial responses to neuronal injury. EMBO Rep.

[4] Takahashi K, Rochford CD, Neumann H (2005). Clearance of apoptotic neurons without inflammation by microglial triggering receptor expressed on myeloid cells-2. J Exp Med.

[5] Wolf EM, Fingleton B, Hasty AH (2022). The therapeutic potential of TREM2 in cancer. Front Oncol.

[6] Nalio Ramos R, Missolo-Koussou Y, Gerber-Ferder Y (2022). Tissue-resident FOLR2(+) macrophages associate with CD8(+) T cell infiltration in human breast cancer. Cell.

[7] Binnewies M, Pollack JL, Rudolph J (2021). Targeting TREM2 on tumor-associated macrophages enhances immunotherapy. Cell Rep.

[8] Esparza-Baquer A, Labiano I, Sharif O (2021). TREM-2 defends the liver against hepatocellular carcinoma through multifactorial protective mechanisms. Gut.

[9] Molgora M, Esaulova E, Vermi W (2020). TREM2 modulation remodels the Tumor Myeloid Landscape enhancing Anti-PD-1 immunotherapy. Cell.

[10] Obradovic A, Chowdhury N, Haake SM (2021). Single-cell protein activity analysis identifies recurrence-associated renal tumor macrophages. Cell.

[11] Park MD, Reyes-Torres I, LeBerichel J (2023). TREM2 macrophages drive NK cell paucity and dysfunction in lung cancer. Nat Immunol.

[12] Yu M, Chang Y, Zhai Y (2022). TREM2 is associated with tumor immunity and implies poor prognosis in glioma. Front Immunol.

[13] Chen X, Zhao Y, Huang Y (2023). TREM2 promotes glioma progression and angiogenesis mediated by microglia/brain macrophages. Glia.

[14] Sun R, Han R, McCornack C (2023). TREM2 inhibition triggers antitumor cell activity of myeloid cells in glioblastoma. Sci Adv.

[15] Zheng J, Wang L, Zhao S et al. TREM2 mediates MHCII-associated CD4 (+) T cell response against gliomas. bioRxiv 202310.1101/2023.04.05.535697. 10.1093/neuonc/noad214 PMC11066911 37941134

[16] Wang XQ, Tao BB, Li B (2016). Overexpression of TREM2 enhances glioma cell proliferation and invasion: a therapeutic target in human glioma. Oncotarget.

[17] Shen X, Burguillos MA, Osman AM (2016). Glioma-induced inhibition of caspase-3 in microglia promotes a tumor-supportive phenotype. Nat Immunol.

[18] Safdar S, Taite LJ (2012). Targeted diazeniumdiolates: localized nitric oxide release from glioma-specific peptides and proteins. Int J Pharm.

[19] Yao S, Zheng M, Wang Z (2022). Self-Powered, Implantable, and wirelessly controlled NO Generation System for Intracranial Neuroglioma Therapy. Adv Mater.

[20] Li C, Zhao B, Lin C (2019). TREM2 inhibits inflammatory responses in mouse microglia by suppressing the PI3K/NF-κB signaling. Cell Biol Int.

[21] Byun JK, Park M, Lee S (2020). Inhibition of glutamine utilization synergizes with Immune checkpoint inhibitor to promote Antitumor Immunity. Mol Cell.

[22] Xue N, Zhou Q, Ji M (2017). Chlorogenic acid inhibits glioblastoma growth through repolarizating macrophage from M2 to M1 phenotype. Sci Rep.

[23] Lisi L, Ciotti GM, Braun D (2017). Expression of iNOS, CD163 and ARG-1 taken as M1 and M2 markers of microglial polarization in human glioblastoma and the surrounding normal parenchyma. Neurosci Lett.

[24] Somasundaram V, Nadhan R (2016). Nitric oxide and reactive oxygen species: clues to target oxidative damage repair defective breast cancers. Crit Rev Oncol Hematol.

[25] Peng H, Nixon K (2021). Microglia Phenotypes Following the Induction of Alcohol Dependence in adolescent rats. Alcohol Clin Exp Res.

[26] Burke NN, Kerr DM, Moriarty O (2014). Minocycline modulates neuropathic pain behaviour and cortical M1-M2 microglial gene expression in a rat model of depression. Brain Behav Immun.

[27] Deng S, Guo A, Huang Z, et al. The exploration of neuroinflammatory mechanism by which CRHR2 deficiency induced anxiety disorder. Prog Neuropsychopharmacol Biol Psychiatry. 2023;110844. 10.1016/j.pnpbp.2023.110844. 10.1016/j.pnpbp.2023.110844 37640149

[28] Kashfi K, Kannikal J, Nath N, Cells. 2021;10(11)10.3390/cells10113194. 10.3390/cells10113194 PMC8624911 34831416

[29] Porta C, Rimoldi M, Raes G (2009). Tolerance and M2 (alternative) macrophage polarization are related processes orchestrated by p50 nuclear factor kappaB. Proc Natl Acad Sci USA.

[30] Takase Y, Kai K, Masuda M (2010). Endoglin (CD105) expression and angiogenesis status in small cell lung cancer. Pathol Res Pract.

[31] Miyata Y, Sagara Y, Watanabe S (2013). CD105 is a more appropriate marker for evaluating angiogenesis in urothelial cancer of the upper urinary tract than CD31 or CD34. Virchows Arch.

[32] Hambardzumyan D, Gutmann DH, Kettenmann H (2016). The role of microglia and macrophages in glioma maintenance and progression. Nat Neurosci.

[33] Carvalho da Fonseca AC, Wang H, Fan H (2014). Increased expression of stress inducible protein 1 in glioma-associated microglia/macrophages. J Neuroimmunol.

[34] Coniglio SJ, Eugenin E, Dobrenis K (2012). Microglial stimulation of glioblastoma invasion involves epidermal growth factor receptor (EGFR) and colony stimulating factor 1 receptor (CSF-1R) signaling. Mol Med.

[35] Wick W, Platten M, Weller M (2001). Glioma cell invasion: regulation of metalloproteinase activity by TGF-beta. J Neurooncol.

[36] Müller A, Brandenburg S, Turkowski K (2015). Resident microglia, and not peripheral macrophages, are the main source of brain tumor mononuclear cells. Int J Cancer.

[37] Hwang SY, Yoo BC, Jung JW (2009). Induction of glioma apoptosis by microglia-secreted molecules: the role of nitric oxide and cathepsin B. Biochim Biophys Acta.

[38] Daws MR, Lanier LL, Seaman WE (2001). Cloning and characterization of a novel mouse myeloid DAP12-associated receptor family. Eur J Immunol.

[39] Schmid CD, Sautkulis LN, Danielson PE (2002). Heterogeneous expression of the triggering receptor expressed on myeloid cells-2 on adult murine microglia. J Neurochem.

[40] Ni JW, Li CX, Chen XW (2022). Triggering receptor expressed on myeloid Cell-2 protects PC12 cells Injury by inhibiting BV2 microglial activation. Neurol India.

[41] Liu PS, Wang H, Li X (2017). α-ketoglutarate orchestrates macrophage activation through metabolic and epigenetic reprogramming. Nat Immunol.

[42] Kieler M, Hofmann M, Schabbauer G (2021). More than just protein building blocks: how amino acids and related metabolic pathways fuel macrophage polarization. Febs j.

[43] Schmid MC, Khan SQ, Kaneda MM (2018). Integrin CD11b activation drives anti-tumor innate immunity. Nat Commun.

[44] Yao Y, Xu XH, Jin L (2019). Macrophage polarization in physiological and pathological pregnancy. Front Immunol.

[45] Murray PJ (2017). Macrophage polarization. Annu Rev Physiol.

[46] Porta C, Riboldi E, Ippolito A (2015). Molecular and epigenetic basis of macrophage polarized activation. Semin Immunol.

[47] Wang N, Liang H, Zen K (2014). Molecular mechanisms that influence the macrophage m1-m2 polarization balance. Front Immunol.

[48] Martinez FO, Gordon S (2014). The M1 and M2 paradigm of macrophage activation: time for reassessment. F1000Prime Rep.

[49] Kou Y, Ji L, Wang H (2017). Connexin 43 upregulation by dioscin inhibits melanoma progression via suppressing malignancy and inducing M1 polarization. Int J Cancer.

[50] He W, Zhu Y, Mu R (2017). A Jak2-selective inhibitor potently reverses the immune suppression by modulating the tumor microenvironment for cancer immunotherapy. Biochem Pharmacol.

[51] Fujiwara Y, Komohara Y, Kudo R (2011). Oleanolic acid inhibits macrophage differentiation into the M2 phenotype and glioblastoma cell proliferation by suppressing the activation of STAT3. Oncol Rep.

[52] Geiß C, Salas E, Guevara-Coto J, et al. Multistability in macrophage activation pathways and metabolic implications. Cells. 2022;11(3). 10.3390/cells11030404. 10.3390/cells11030404 PMC8834178 35159214

