# Supplementary material for: Knockdown of trem2 promotes proinflammatory microglia and inhibits glioma progression via the JAK2/STAT3 and NF-κB pathways
Source: Cell Commun Signal. 2024 May 15;22:272. doi: 10.1186/s12964-024-01642-6 (PMC11094905; doi:10.1186/s12964-024-01642-6)
Supplement: Supplementary file 1 — Supplementary Material 1 [file 12964_2024_1642_MOESM1_ESM.docx]

**Fig1a**


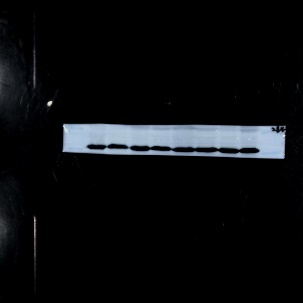

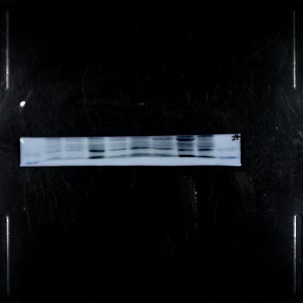


**Fig1g**


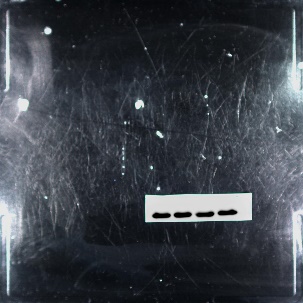

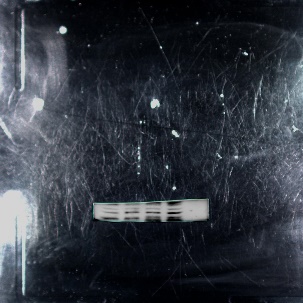


**Fig1h**

**
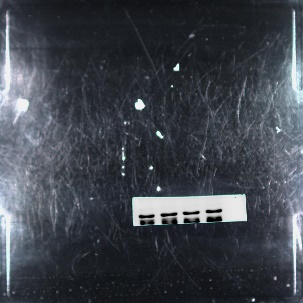

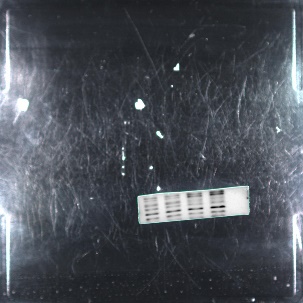
**

**Fig1i**

**
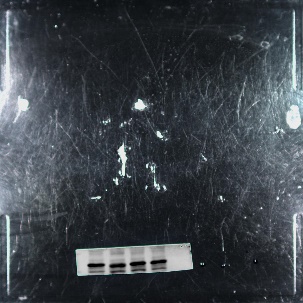

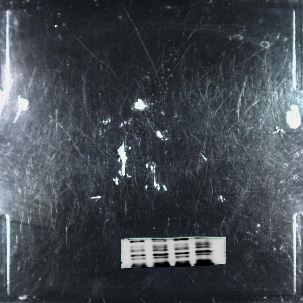
**

**Fig1j-U251**

**
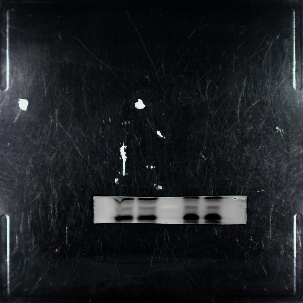

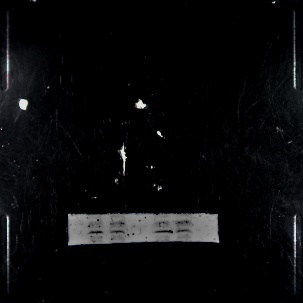
**

**Fig1j-U87**

**
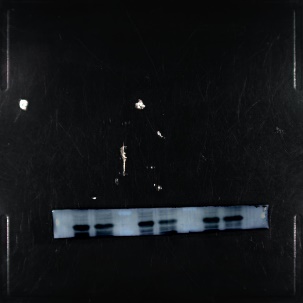

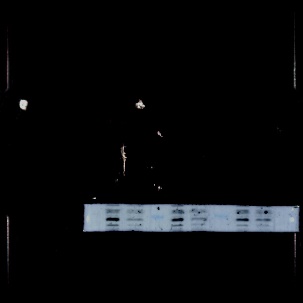
**

**Fig1k-U251**

**
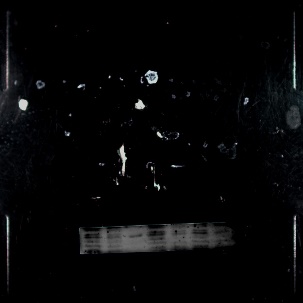

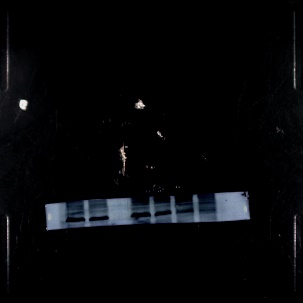
**

**Fig1k-U87**

**
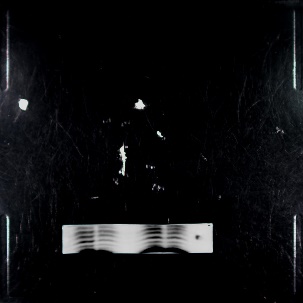

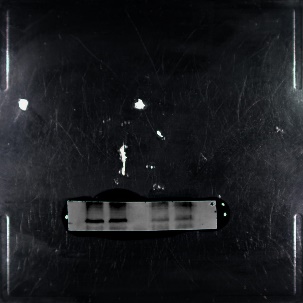
**

**Fig1l**

**
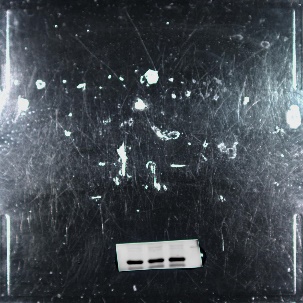
** **
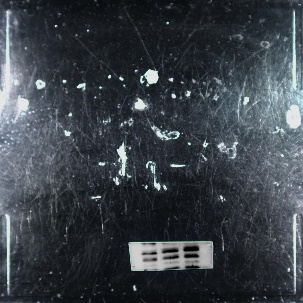
**

**Fig1m**

**
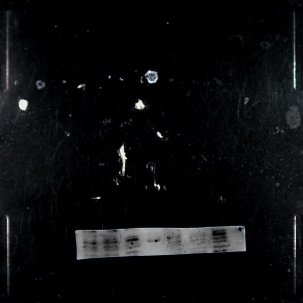
** **
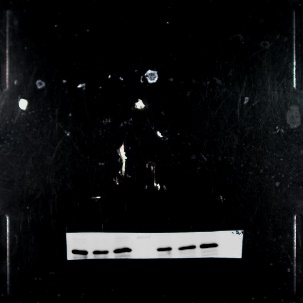
**

**Fig2a**

**
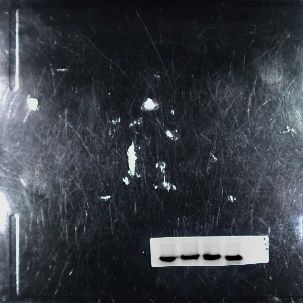
** **
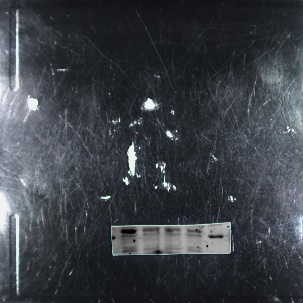
**

**Fig2b**

**
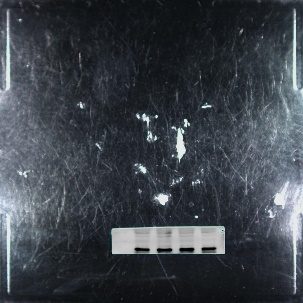
** **
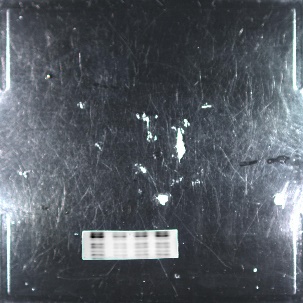
**

**Fig2m-U87**


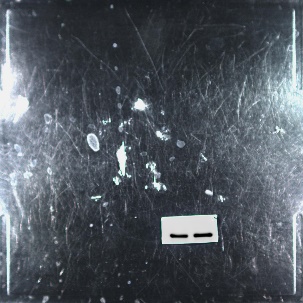

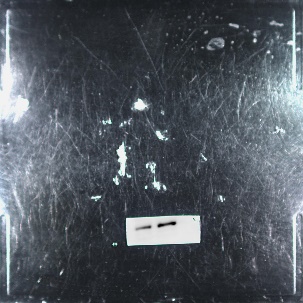

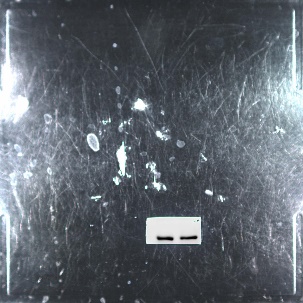

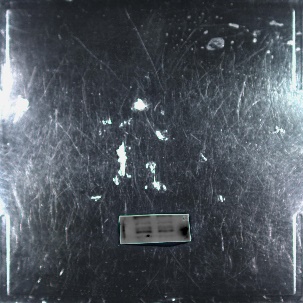

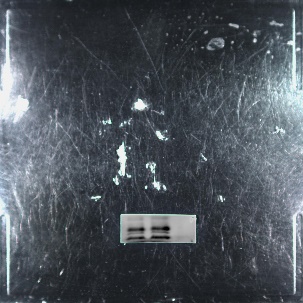


**Fig2m-U251**


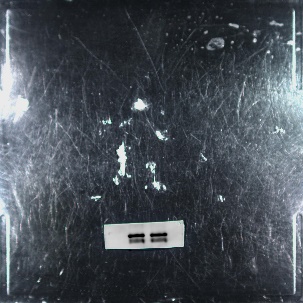

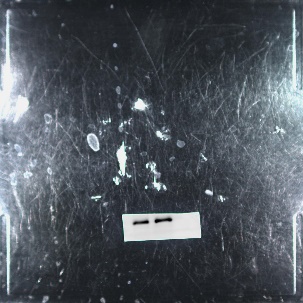

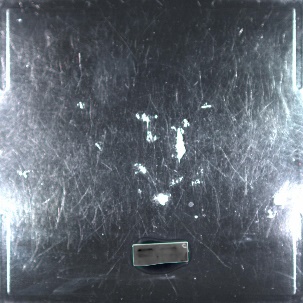

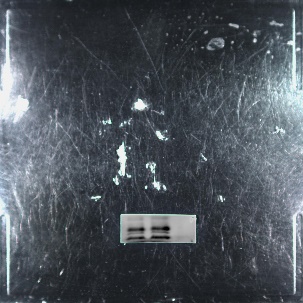

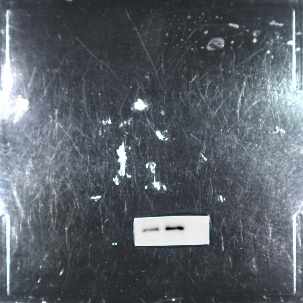


**Fig3b**

**
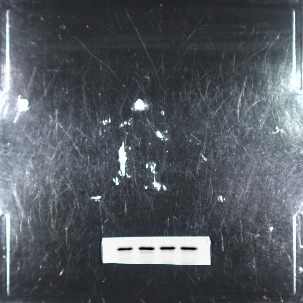
** **
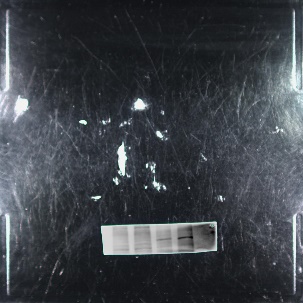
**

**Fig3c**

**
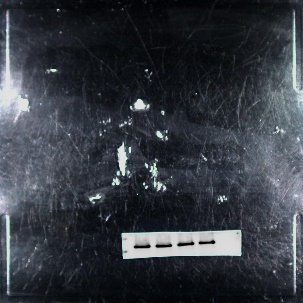
** **
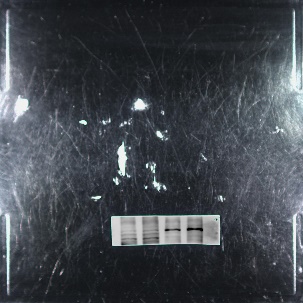
**

**Fig3e**

**
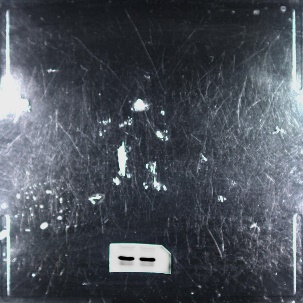
** **
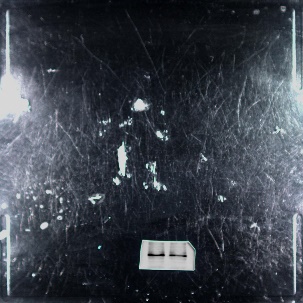
**
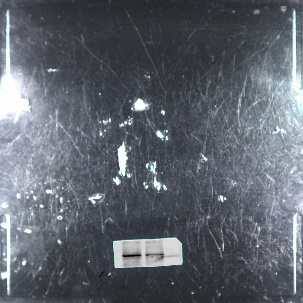

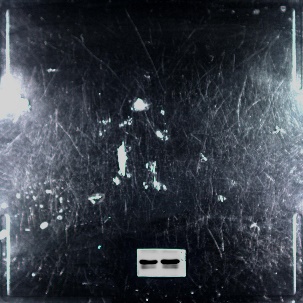


**Fig3h-U87**


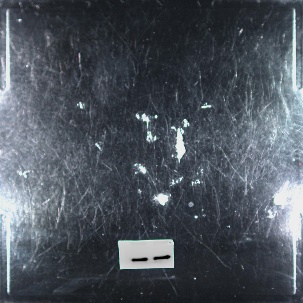

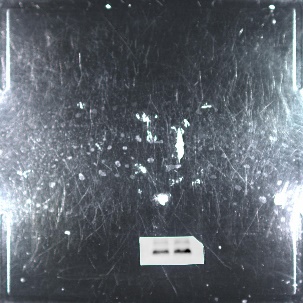

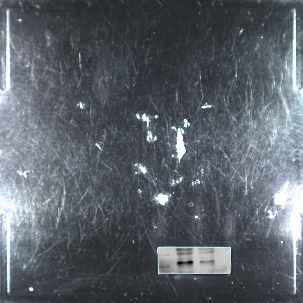


**Fig3h-U251**


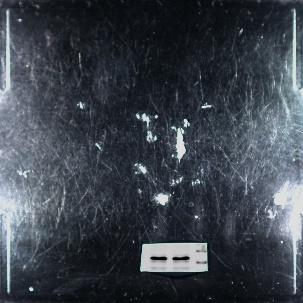

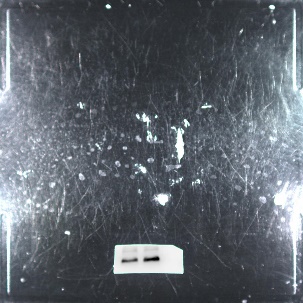

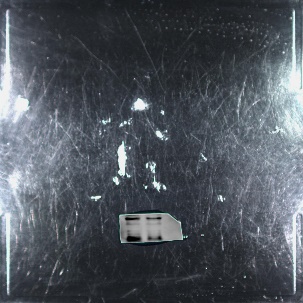


**Fig3o**

**
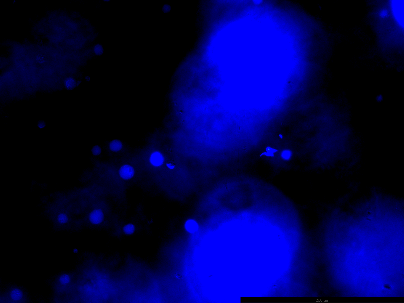
** **
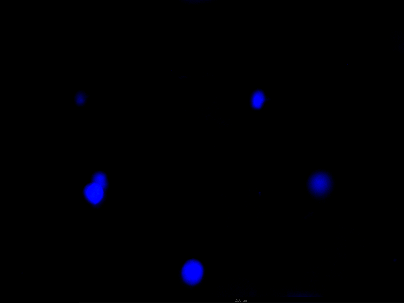

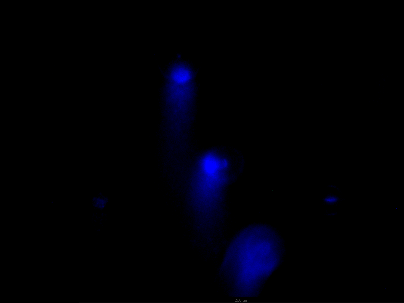
**

**Fig5p**

**
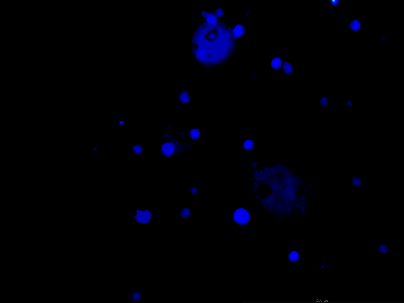
** **
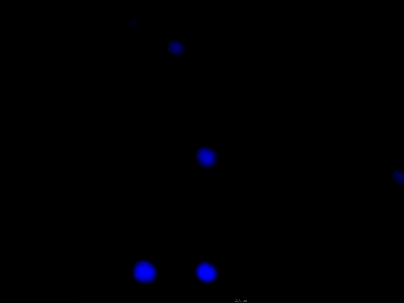
**
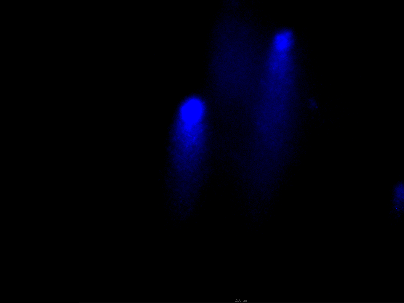


**Fig4a**

**
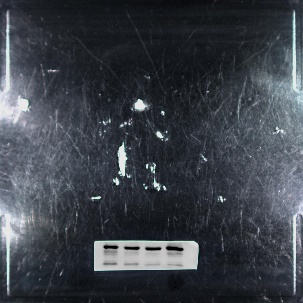
** **
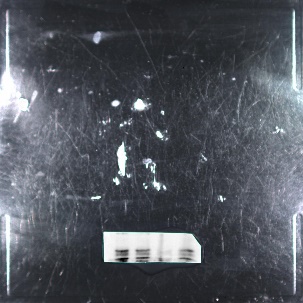
**
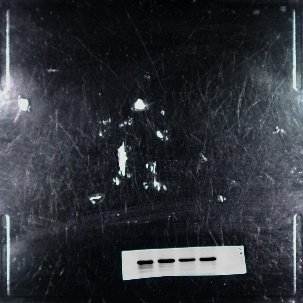

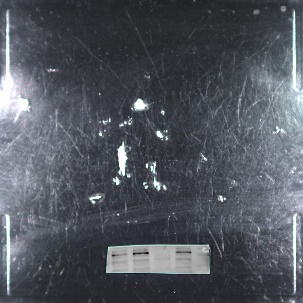


**Fig4b-hmc3**

**
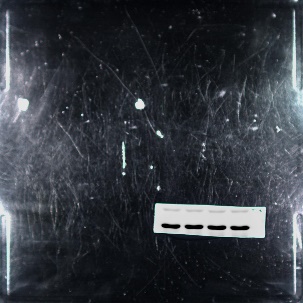
** **
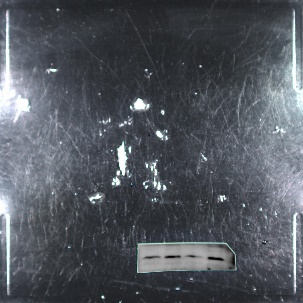
**
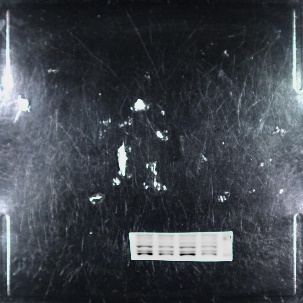

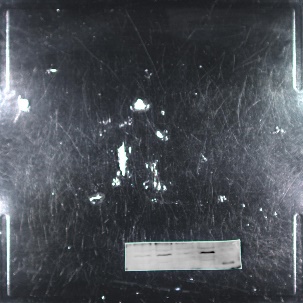

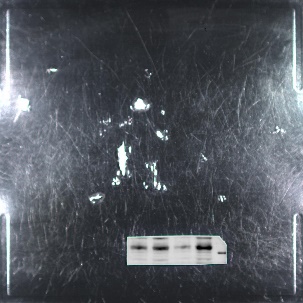


**Fig4b-BV2**

**
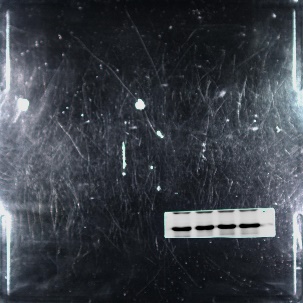
** **
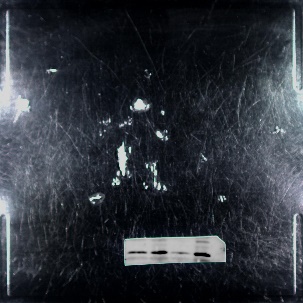
**
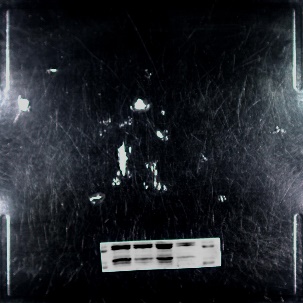

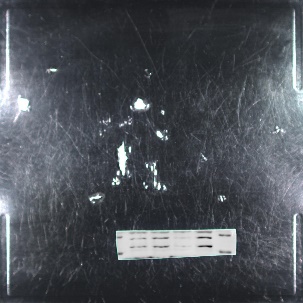
**
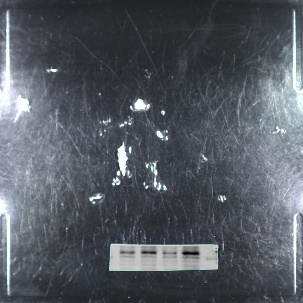
**

**Fig4d**

**
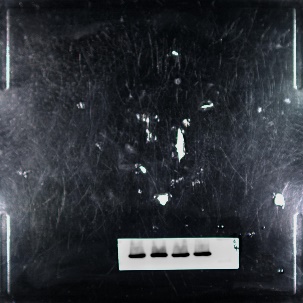
** **
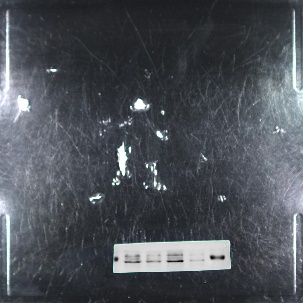
**
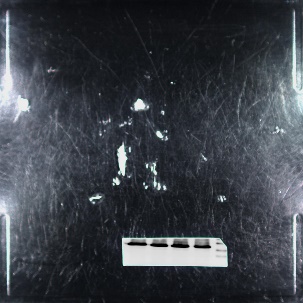

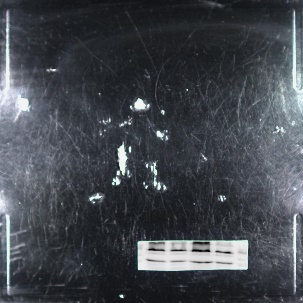


**Fig4e-hmc3**

**
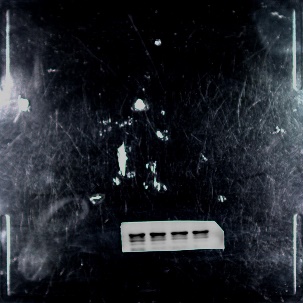
** **
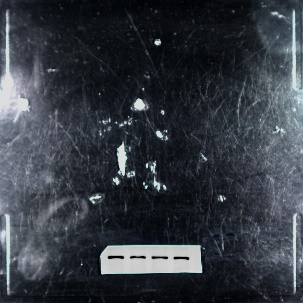
**
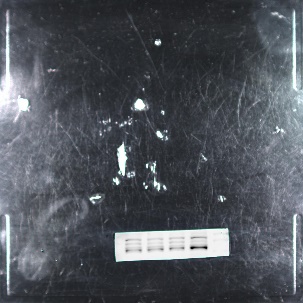


**Fig4e-BV2**


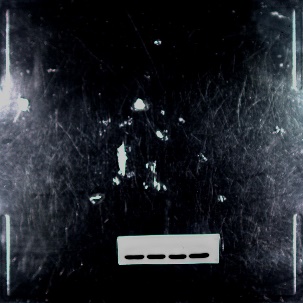
 **
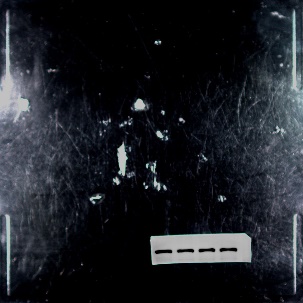
**
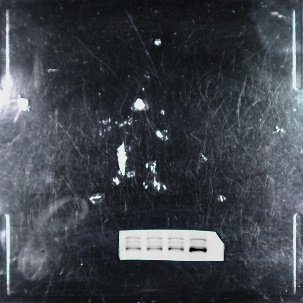


**Fig4f-hmc3**

**
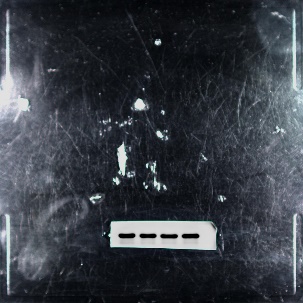
** **
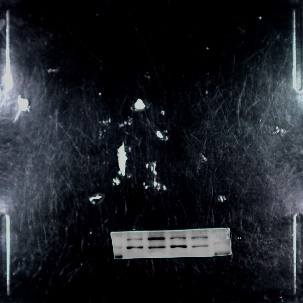
**
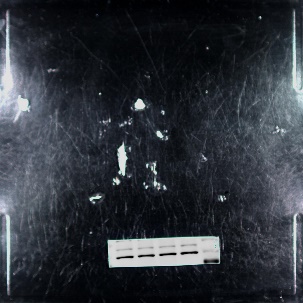


**Fig4f-BV2**

**
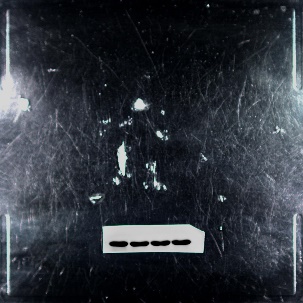
** **
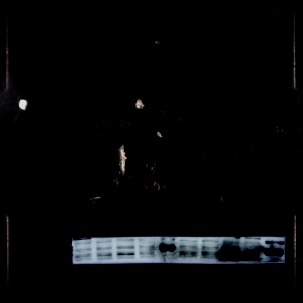
**
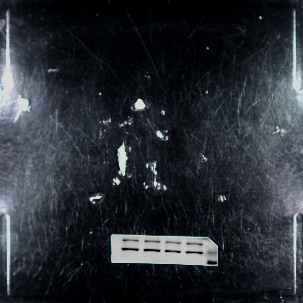


**Fig4g-hmc3**

**
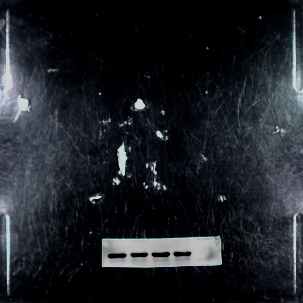
** **
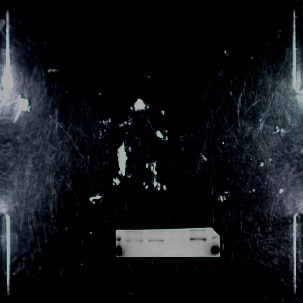
**
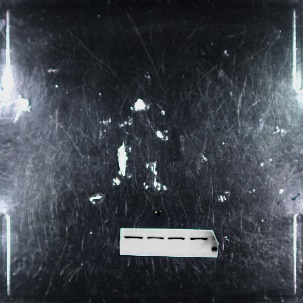


**Fig4g-BV2**

**
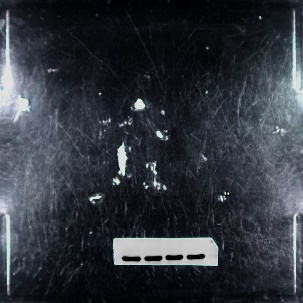
** **
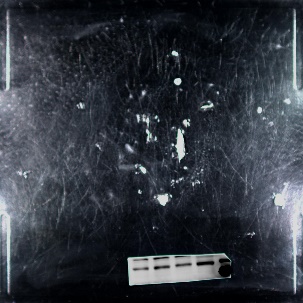
**
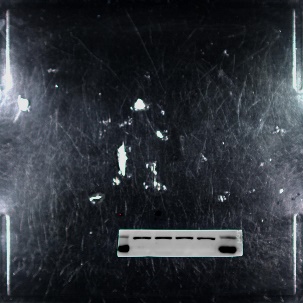


**Fig5f-U251**

**
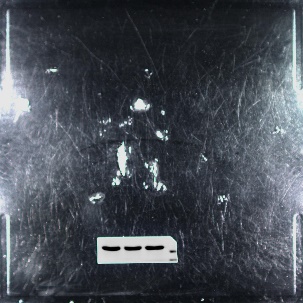

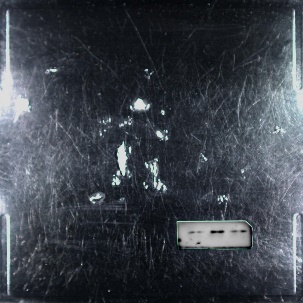

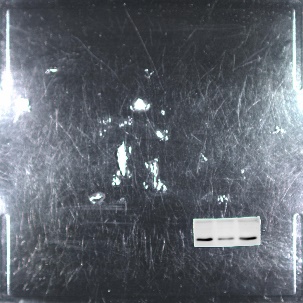
**

**Fig5f-gl261**

**
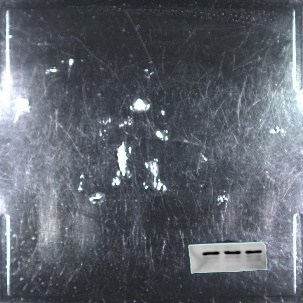
** **
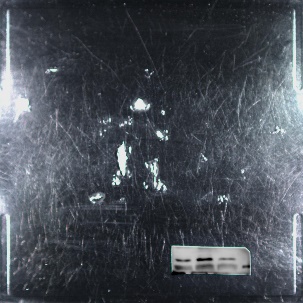
**
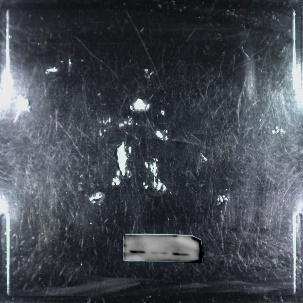


**Fig5h-U251**

**
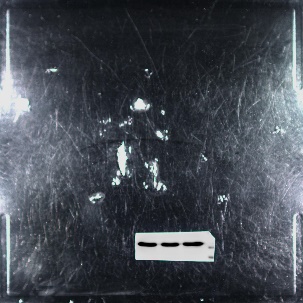
** **
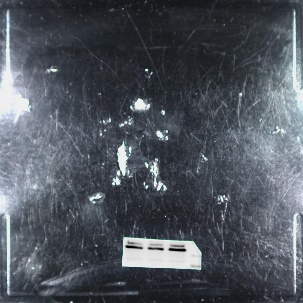
**
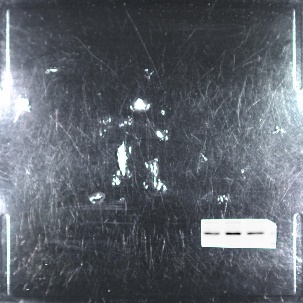

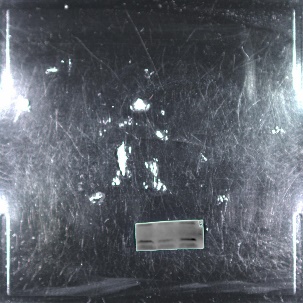


**Fig5h-gl261**
